# The Internalization Pathways of Liposomes, PLGA, and Magnetic Nanoparticles in Neutrophils

**DOI:** 10.3390/biomedicines12102180

**Published:** 2024-09-25

**Authors:** Anastasiia Garanina, Daniil Vishnevskiy, Anastasia Chernysheva, Julia Malinovskaya, Polina Lazareva, Alevtina Semkina, Maxim Abakumov, Victor Naumenko

**Affiliations:** 1 Laboratory of Biomedical Nanomaterials, National University of Science and Technology «MISIS», 119049 Moscow, Russia; abakumov1988@gmail.com; 2 Department of Medical Nanobiotechnology, N.I. Pirogov Russian National Research Medical University, 117997 Moscow, Russia; vishnevskiy.daniil.andreevich@gmail.com (D.V.); polizara604@gmail.com (P.L.); alevtina.semkina@gmail.com (A.S.); 3 V. Serbsky National Medical Research Center for Psychiatry and Narcology, 119034 Moscow, Russia; aachernysheva512@gmail.com (A.C.); naumenko.vict@gmail.com (V.N.); 4 Drug Delivery Systems Laboratory, D. Mendeleev University of Chemical Technology of Russia, 125047 Moscow, Russia; j.malinowskaya@gmail.com

**Keywords:** nanoparticles, neutrophils, endocytosis, cell-based delivery system, cancer treatment

## Abstract

Background/Objectives: Neutrophils are emerging as promising candidates for cell-based nanodrug delivery to tumors due to their unique biological properties. This study aims to investigate the mechanisms of nanoparticle internalization by neutrophils, specifically focusing on liposomes, poly(lactic-co-glycolic acid) (PLGA), and magnetite nanoparticles. Understanding these mechanisms could enhance the efficiency of neutrophil-based nanodrug delivery for cancer treatment. Methods: Neutrophils were isolated from the peripheral blood of mice bearing 4T1 mammary adenocarcinoma. Confocal microscopy, transmission electron microscopy, and flow cytometry were employed to evaluate the uptake of liposomes, PLGA, and magnetite nanoparticles by neutrophils. The effects of cultivation conditions, such as the presence or absence of plasma in the growth medium, were also examined. Additionally, the roles of immunoglobulins (IgG/IgM) and cell surface receptors (Fc and scavenger receptors) in nanoparticle internalization were explored. Results: All types of nanoparticles were successfully internalized by neutrophils, though the mechanisms of uptake varied. Plasma presence in the medium significantly influenced nanoparticle binding, particularly for PLGA nanoparticles. Internalization of PLGA nanoparticles was found to depend on the presence of IgG/IgM in the medium and Fc receptors on neutrophil surfaces, while scavenger receptors were not involved. Conclusions: Understanding the distinct endocytosis pathways for different nanoparticles can improve the efficacy of neutrophil loading with nanodrugs, potentially advancing the development of neutrophil-based cancer therapies. The findings underscore the importance of the extracellular environment in modulating nanoparticle uptake.

## 1. Introduction

Nanomedicine is still a growth area of science, in particular nanotechnology [1]. Advances in nanotechnology enable the production of nanoparticles (NPs) with desired chemical and physical properties that can be used for drug delivery [2]. By fine-tuning parameters such as size, shape, and surface coating of the nanocarrier, it has become possible to change the pharmacokinetics and pharmacodynamics of the drug, increasing the efficiency of its targeted delivery and accumulation as well as reducing side effects. Creation of nanodrugs for cancer treatment is actively studied and developed nowadays. It is known that the blood and lymphatic vessels of a tumor significantly differ from those in normal tissues, which can be used for effective nanodrug delivery to malignant cells. It was assumed that a defective vascular barrier in the tumor microenvironment is permeable to nanosized particles, and increased interstitial pressure in the tumor prevents the removal of NPs through the lymphatic system. These specific properties of tumor vessels were described as the EPR effect (enhanced permeability and retention). Although the EPR strategy has shown effectiveness in treating tumors in animals, many nanomedicines have failed in translation into the clinic [2,3]. In this regard, new approaches are being developed for nanodrug delivery to tumors. One of them is the use of nanodrug cell-based delivery systems (NCBDs) [4,5]. Different types of cells have been proposed as NCBDs, such as mesenchymal stem cells, leukocytes, and red blood cells [6]. Peripheral blood neutrophils are considered the most promising candidates due to their large numbers (65–80% of circulating leukocytes) and their ability to cross the vascular barrier and to migrate over long distances in the body in response to the chemokines produced in tumor lesions [7]. Nevertheless, the success of using neutrophils as NCBDs largely depends on the efficiency of the cell loading with NPs. Despite the large number of studies describing the interactions of neutrophils with NPs [8,9,10], the molecular mechanisms of NP uptake remain poorly understood. It is supposed that the basic way for the NP internalization by leukocytes is phagocytosis [11]. In this case, the optimal particle size for engulfment is about 2–3 µm [12,13]. However, the size of NPs usually varies in the range from 10 to 500 nm. It cannot be ruled out that NPs form larger conglomerates on the cell membrane, accessible for phagocytosis [14]. An alternative hypothesis is that non-phagocytic types of endocytosis, such as clathrin- or caveolin-mediated, clathrin/caveolin-independent (clathrin-independent carriers/GPI-AP-enriched early endosomal compartment (CLIC–GEEC), RhoA-dependent, Arf6-associated, etc.), and macropinocytosis, are involved in the uptake process. Clathrin- and caveolin-dependent types of endocytosis are effective for capturing NPs in the range of 25–30 nm [14,15] but are also capable of entrapping NPs approximately 100 and 60 nm in size, respectively [16]. Internalization of larger NPs is possible via macropinocytosis, during which the capture of extracellular fluid occurs, and the vesicles (macropinosomes) ranging in size from 250 nm to 5 μm in diameter are formed [17]. 

The mechanism of NP uptake by cells can be influenced not only by the size but also by the shape of NPs. Xie et al. demonstrated that different gold nanostars, nanorods, and nanotriangles coated with methylpolyethylene glycol penetrated macrophages RAW264.7 through clathrin-mediated endocytosis [18]. Nanorods could be internalized via caveolin- or lipid raft-mediated endocytosis, while triangles could be internalized via additional actin and dynamin-dependent pathways (possibly phagocytosis and/or macropinocytosis). Ding et al. found that spherical gold NPs with a diameter less than 45 nm as well as rod-shaped NPs are engulfed by a clathrin-mediated endocytosis [19]. The uptake of star-shaped NPs occurs by both the clathrin- and caveolin-mediated endocytosis types. 

Other important NP characteristics that affect the pathway of their uptake by cells are chemical composition and surface modification. Albumin-based NPs (for example, human serum albumin (HSA) or bovine serum albumin (BSA) NPs), which can be charged either positively or negatively depending on the chemical modification or loaded drugs, enter the cells mainly by clathrin-mediated endocytosis [20]. Nevertheless, electronegative plasmid-loaded HSA-based NPs were found to be internalized by the caveolin-mediated pathway. The alginate-based NP uptake mechanism depends on their size. NPs with sizes less than 120 nm penetrate the cells by clathrin-mediated type of endocytosis, with sizes about 420 nm—by caveolin-mediated one—and 730 nm sized NPs—by macropinocytosis. Wang et al. found that polystyrene NPs are internalized by clathrin-mediated endocytosis, regardless of their size [21]. Platel et al. revealed that poly(lactic-co-glycolic acid) (PLGA) positively charged NPs are generally taken up by clathrin-mediated endocytosis, while negatively charged ones are internalized by clathrin-/caveolin-independent pathways [22]. Liposomes were shown to enter the cells mainly by clathrin-mediated endocytosis [23]. Nevertheless, the caveolin-mediated pathway and macropinocytosis can also occur after lipid NP surface modifications [24]. Iron oxide NPs (IONPs) enter the cells by caveolin-mediated endocytosis in most cases [25]; however, the modification of their surface with dimercaptosuccinate changes the way of internalization to the clathrin-mediated pathway [26] or even to macropinocytosis [27] depending on cell type. Finally, the uptake of gold NPs depends not only on their shape, as described above [19], but also on surface decoration. Coating of NPs with citrate leads to their engulfment by macropinocytosis and by clathrin- or caveolin-mediated endocytosis, while modification with polyethylene glycol (PEG) results in NP internalization by clathrin- and caveolin-mediated endocytosis but not by macropinocytosis [14].

For neutrophils, it was shown that they engulf large NPs more efficiently than small ones (20–200 nm) [28]. Moreover, these cells preferentially uptake elongated NPs [29]. And finally, in human serum, neutrophils were found to internalize poly(styrene) NPs and liposomes less effectively than PLGA NPs [28]. However, the molecular mechanism of this difference is not clear. 

Understanding the mechanisms of NP endocytosis by neutrophils is important not only for increasing cell loading with drugs but also for assessing the functional activity of neutrophils, which can be affected by different types of endocytosis [30]. Clathrin-dependent mechanism plays an important role in the neutrophil response to various stimuli. For example, it leads to neutrophil priming—a state in which the cell’s protective arsenal is not yet used but is already put on high alert. This type of endocytosis is also involved in cell polarization [31] as well as in the regulation of cell inhibition processes. Caveolin-dependent endocytosis is associated with neutrophil adhesion and migration [32]. The role of macropinocytosis in neutrophils is poorly studied [33], and it is associated with the mechanisms of cell migration. 

Thus, features of neutrophil interaction with NPs and the internalization mechanisms of the latter depend on the certain type of NP. However, currently available data are very scattered. Previously, we presented a comparative analysis of neutrophil interaction with three NP types (liposomes, PLGA, and magnetite nanoparticles) [34], which have been approved for cancer treatment [35,36].

In this follow-up study, the mechanisms of NP internalization by neutrophils were investigated in detail. Cells isolated from the peripheral blood of 4T1-bearing mice were used since the role of neutrophils in NP delivery to this tumor model has been previously demonstrated [34]. All NP types were shown to be uptaken by neutrophils in vitro. After 1 h of co-incubation, they could be located both in the cell cytoplasm and inside vesicles that were neither early nor late endosomes. However, the ways of NP endocytosis differed, wherein the cultivation conditions, such as plasma presence or absence in a culture medium, strongly affected neutrophil binding with NPs. This was especially pronounced for PLGA NPs. Understanding the mechanisms of NP interactions with neutrophils could help increase the effectiveness of neutrophil loading with nanodrugs for cancer treatment. 

## 2. Materials and Methods

### 2.1. Nanoparticles’ Synthesis

The synthesis of NPs is described in detail in [34]. It is also briefly presented in Supplementary Information.

### 2.2. Animals and Tumor Model

All experiments conducted on animals were approved by the N.I. Pirogov Russian National Research Medical University bioethical committee (protocol # 16/2021). BALB/c mice were obtained from the Andreevka Animal Center (Andreevka, Russia). Seven- to nine-week-old female animals with weights of 20–22 g were included in the study. 4T1 (mouse breast cancer cells) were purchased from the American Type Culture Collection (ATCC, Manassas, VA, USA). The cells were cultured in RPMI-1640 medium (gibco, New York, NY, USA) with 10% fetal bovine serum (FBS, gibco) and 2 mM L-glutamine (gibco). A total of 1 × 10^6^ cells were subcutaneously (s.c.) implanted in the right hind flank to establish the tumor.

### 2.3. Neutrophils Isolation from the Blood of Tumor-Bearing Mice

The neutrophil isolation protocol is described in [34]. Briefly, the blood was collected on the 6th day after the 4T1 cell implantation through cardiac puncture. Syringes containing 2% EDTA were used. Later, the blood was centrifuged at 400× *g* for 20 min at room temperature. The separated plasma was transferred to another tube for the following experiments. The obtained cellular component was mixed with 6% Ficoll 400 (Serva-Feinbiochemica GmbH & Co, Heidelberg, Germany) solution in 0.9% NaCl (1:2, *v*:*v*), left for 1 h at room temperature, and then washed twice in a PBS/0.2% EDTA solution. The pure neutrophil fraction was isolated by the gradient method using Percoll Plus (Cytiva GE Healthcare’s). For this, the cellular component was layered on a Percoll 60%/80% gradient and centrifuged at 1500× *g* for 45 min at room temperature. The fraction of neutrophils was collected at the boundary between Percoll 60% and 80%, washed twice, and resuspended in RPMI-1640 medium. A TC20 Automated Cell Counter (Bio Rad, Hercules, CA, USA) was used to count the cells and analyze their viability after staining with trypan blue dye. To assess the purity of the obtained cell fraction, it was stained with fluorophore-conjugated Ly6G BV421 (Clone 1A8), CD45 FITC (Clone 30-F11), and CD11b PE (Clone M1/70) (all from Biolegend, San Diego, CA, USA, dilution 1:100) antibodies for 30 min at 4 °C, washed, and analyzed using a MoFlo flow cytometer (Beckman Coulter; Miami, FL, USA). It was revealed that the viability of isolated neutrophils was 97%, and the population purity was (98.2 ± 0.75)%.

### 2.4. Study of Intracellular Nanoparticles’ Localization

#### 2.4.1. Immunocytochemical Staining

Isolated neutrophils were seeded on coverslips at a concentration of 5 × 10^6^ cells/mL, incubated for 1 h in the presence of NPs, washed with DPBS (pH 7.2–7.4, gibco), fixed with 4% formalin for 15 min, and washed again. The final concentration of NPs in the culture medium was as follows: 100 μg/mL (by iron) and 0.4 μg/mL (by Cy5) for MNPs-Cy5, 1 mg/mL (by PLGA) and 0.72 μg/mL (by Cy5) for PLGA-Cy5, and 1.68 mg/mL (by lipids) and 1.1 μg/mL (by DiD) for liposomes-DiD. To permeabilize the membranes, the cells were placed in a 0.2% solution of Triton X100 detergent in DPBS for 6–7 min and then washed with DPBS 3 times for 5 min. Later, the samples were left in a 3% solution of bovine serum albumin (BSA) in DPBS for 45 min at room temperature and placed in the primary antibodies (1:100 in DPBS) for 1 h at room temperature in a humid chamber. After this, they were washed three times with DPBS for 5 min, placed in secondary antibodies (1:700 in DPBS) under similar conditions, and washed again. The nuclei of cells were counterstained with DAPI (0.1 μg/mL) (Sigma, New York, NY, USA) for 10 min. The samples were then embedded in Dako Mounting Medium (Agilent Technologies, Santa Clara, CA, USA) and analyzed using the confocal microscope Nikon Eclipse Ti-E A1R MP (Apo LWD 40x/1.15 S water immersion objective (numerical aperture = 1.15; Nikon, Japan)). Post-processing of the obtained images was carried out using NIS-Elements AR software V.5.15. Monoclonal rabbit antibodies to early (Rab5, EEA1) and late (Rab7) endosomes (Thermo Fisher Scientific, Waltham, MA, USA) were used as primary antibodies. The secondary antibodies were goat anti-rabbit IgG conjugated to Alexa Fluor 488 (Thermo Fisher Scientific).

#### 2.4.2. Transmission Electron Microscopy

To study the intracellular localization of NPs using TEM, MNPs-Cy5 were selected. After incubation of isolated neutrophils with this type of NPs (at a concentration of 100 μg/mL (by iron)) for 1 h, the cells were fixed with 2.5% glutaraldehyde with 2% formaldehyde in 0.1 M PBS (pH 7.2) for 1 h. After fixation, the samples were washed with PBS 2 times for 15 min each, and then post-fixation was carried out in 1% OsO_4_ for 1 h in the darkness. Later, the cells were sequentially placed in 50% ethanol for 5 min, 70% ethanol for 5 min, 80% ethanol for 3 × 10 min, 96% ethanol for 1 h, a 3:1 96% ethanol:Epon mixture for 1 h, a 96% ethanol:Epon 1:1 mixture for 2 h, a 96% ethanol:Epon 1:3 mixture overnight, and Epon for 7 h, embedded into Epon, and left in a thermostat for polymerization for 24 h at 37 °C and 48–72 h at 60 °C. The Epon mixture contained Epon 812, DDSA, MNA, and polymerization catalyzer DMP-30 (1 mass%, Fluka Chemikals, Morris Plains, NJ, USA). The 70 nm sections were prepared on an LKB II ultramicrotome using a diamond knife and counterstained with a 1.5% aqueous solution of uranyl acetate at room temperature in a wet chamber. The obtained samples were examined using a transmission electron microscope (TEM) JEOL JEM-1400 (120 kV) (JEOL Ltd., Tokyo, Japan). Neutrophils incubated in the medium without the addition of MNPs-Cy5 were taken as a control. 

### 2.5. Dynamics of Nanoparticles’ Interaction with Neutrophils In Vitro

The protocol was described previously in [34]. Briefly, isolated neutrophils were seeded on coverslips at a concentration of 5 × 10^6^ cells/mL in RPMI-1640 medium with collected blood plasma (1:1, *v*:*v*) or without it. NPs were added to the cells at the final concentrations of 100 μg/mL (by iron) and 0.4 μg/mL (by Cy5) for MNPs-Cy5, 1 mg/mL (by PLGA) and 0.72 μg/mL (by Cy5) for PLGA-Cy5, and 1.68 mg/mL (by lipids) and 1.1 μg/mL (by DiD) for liposomes-DiD. Cells were cultivated with NPs for 10–120 min at 37 °C. The obtained samples were washed 3 times with DPBS, stained with Ly6G BV421 (Clone 1A8, Biolegend, dilution 1:100) antibodies for 30 min, washed 3 times with DPBS, fixed with 4% formalin in DPBS over 15 min, placed in Dako Mounting Medium, and studied with the confocal microscope Nikon Eclipse Ti-E A1R MP (Plan Apo 20x/0.75 Dic N objective (numerical aperture = 0.75); Nikon, Japan). Neutrophils incubated without NPs or with the addition of free Cy5 dye (in corresponding concentrations of 0.4 μg/mL and 0.72 μg/mL) were taken as controls (data are presented in [34]). The accumulation of NPs in the neutrophils was analyzed by calculating the average intensity of the fluorescence signal from the NPs in cells using the NIS-Elements AR software V.5.15. To identify neutrophils, binary masks were applied to the Ly6G signal in the images. The masks, representing clusters of neutrophils, were manually segmented. Following this, the masks were used to define distinct regions of interest (ROIs). The fluorescence intensity of the NPs within each ROI was measured, with the intensity of control cells used as the baseline (zero). For each type of NP, 130 neutrophils were analyzed.

### 2.6. Endocytosis Mechanisms’ Investigation by Inhibitory Assay

Isolated neutrophils were seeded on a confocal 96-well plate (µ-Plate 96 Well Black, ibidi) at a concentration of 1.5 × 10^6^ cells/mL in RPMI-1640 medium containing blood plasma (1:1, *v*:*v*) or not and left for 1 h at 37 °C. Endocytosis inhibitors were then added to the neutrophils and incubated for 1 h. The following inhibitor concentrations were used: chlorpromazine (aminazine, Valenta, Russia)—40 μM; MβCD (Sigma, New York, NY, USA)—5 mM; nystatin (Sigma)—50 μM; cytochalasin-D (Sigma)—30 μM; and amiloride (Sigma)—1 mM. Later, the cells were washed twice with HBSS, and solutions of the studied NPs in DPBS (at concentrations indicated above) were added to them for 1 h. All studies were performed in triplicate. Subsequently, neutrophils were washed with HBSS, stained with fluorophore-conjugated antibodies Ly6G BV421 (Clone 1A8, Biolegend) for 30 min with two subsequent washes with HBSS solution, and fixed with 4% formalin for 15 min. The obtained samples were analyzed using a confocal microscope Nikon Eclipse Ti-E A1R MP (Plan Apo 20x/0.75 Dic N objective (numerical aperture = 0.75); Nikon, Tokyo, Japan). The accumulation of NPs in the neutrophils was analyzed by calculating the average intensity of the fluorescence signal from the NPs in cells using the NIS-Elements AR software V.5.15, as described above. During the analysis, 50 neutrophils were counted for each NP type.

### 2.7. The Role of Neutrophil Receptors and Serum Factors during the Nanoparticles’ Uptake In Vitro

Isolated neutrophils were seeded in black 96-well culture plates for fluorescence microscopy (ibidi) at a concentration of 1.5 × 10^5^ cells per well. Initially, all cells were cultured in RPMI-1640 medium. To identify the role of Fc receptors in the NP uptake, cells were pre-incubated with antibodies to CD16/32 (Purified Rat Anti-Mouse CD16/CD32, Clone 93, Biolegend, San Diego, CA, USA, 1:100 in DPBS/0.2% EDTA solution) for 10 min at 37 °C. To assess the role of scavenger receptors, neutrophils were incubated in the presence of polyinosinic acid (Poly(I), Sigma, 1 μg/mL) for 30 min. After this, the content of the wells was replaced. Growth medium containing blood plasma (1:1, *v*:*v*) and PLGA-Cy5 NPs at a concentration of 1 mg/mL (by PLGA) was added to the cells for 1 h. In addition, to identify the role of IgG/IgM in the NP uptake by neutrophils, cells were cultured with PLGA-Cy5 NPs in a medium supplemented with serum from SCID mice (animals with a genetic immunodeficiency characterized by an almost complete absence of functional B and T lymphocytes) for 1 h. SCID mice serum was kindly provided by Abakumova T.O. from N.I. Pirogov Russian National Research Medical University. Neutrophils incubated with NPs in a medium without plasma, as well as cells cultured in a medium both without plasma and without PLGA-Cy5 NPs, were taken as controls. Then, the cells were washed with a DPBS/0.2% EDTA solution, fixed with 1% formalin overnight, washed again, and stained with fluorophore-conjugated antibodies Ly6G BV421 (Clone 1A8), CD45 FITC (Clone 30-F11), and CD11b PE (Clone M1/70) (all from Biolegend, dilution 1:100) for 30 min at 4 °C. Later, neutrophils were washed; part of the cell samples were dissociated using a TrypLE solution (gibco) and put in DPBS. The obtained samples were analyzed by a MoFlo flow cytometer (Beckman Coulter; Miami, FL, USA) and Nikon Eclipse Ti-E A1R MP confocal microscope using a Plan Apo 20x/0.75 Dic N objective (NA = 0.75; Nikon, Tokyo, Japan). The Summit V5.2.0.7477 software was used for processing data from the flow cytometer. The images from the confocal microscope were processed using the NIS-Elements AR software V.5.15, as described above. All experiments were performed in at least three independent replicates. 

### 2.8. Statistics

The statistical analysis was performed in GraphPad Prism 9. To compare the normally distributed samples, we used an unpaired *t*-test. *p* values < 0.05 were considered to be significant.

## 3. Results

### 3.1. Nanoparticles’ Accumulation in Neutrophils In Vitro

The characteristics of the studied nanoparticles (NPs)—liposomes-DiD, PLGA-Cy5, and magnetic NPs-Cy5 (MNPs)—were described in detail earlier [34]. Briefly, MNPs-Cy5 had a hydrodynamic size (HS) of 35 nm and a ζ-potential of (−26 ± 2) mV, PLGA-Cy5 NPs—HS of 100–120 nm and a ζ-potential of (−23 ± 9) mV, and liposomes-DiD—HS of about 120 nm and a ζ-potential of (−2.6 ± 19.4) mV (in PBS) (Figure S1, [37]). MNPs-Cy5 and liposomes-DiD remained stable in a growth medium containing 10% serum, whereas PLGA-Cy5 formed conglomerates. All NPs were spherical in shape. It was also shown previously that investigated NPs were able to interact with blood neutrophils isolated from the 4T1 tumor-bearing mice. Moreover, NPs did not show toxicity and caused a minimal activation effect on neutrophils.

**Figure 1:**
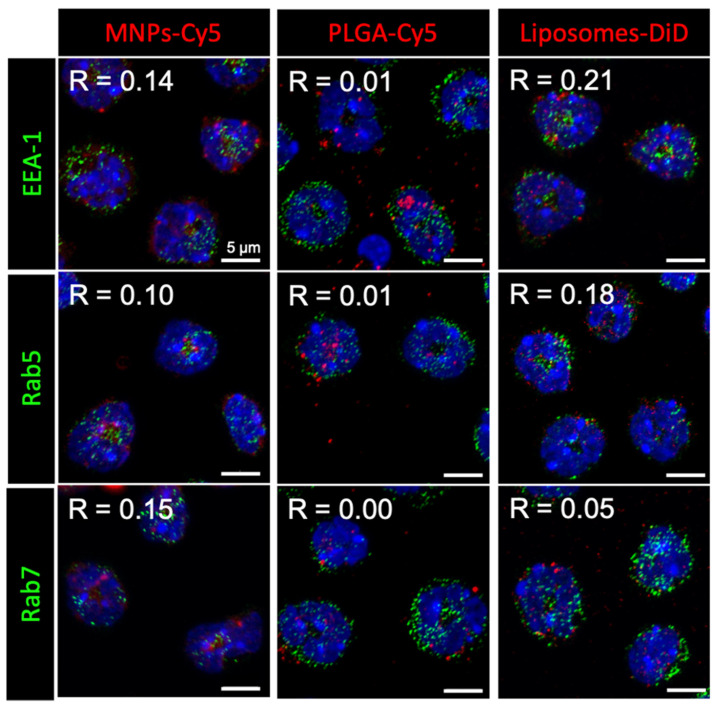
Nanoparticle (red) localization in neutrophils blood isolated from the 4T1 tumor-bearing mice after 1 h of co-incubation. Immunocytochemical staining with antibodies to the proteins EEA-1, Rab5, and Rab7 (green), cell nuclei are stained with DAPI (blue), confocal microscopy, Pearson correlation coefficient (R) values between the endosome markers and NPs are shown.

Here, we approached the study of NP interaction with neutrophils in more detail. Investigation of the NP intracellular localization after 1 h incubation with isolated neutrophils showed that none of the NP types accumulated in early (EEA-1 or Rab5 positive) or late (Rab7 positive) endosomes (Figure 1). Moreover, NP conglomerates had different sizes inside the cells: liposomes-DiD accumulated in the form of clusters smaller than or similar in size to endosomes, MNPs-Cy5 in larger ones, and PLGA-Cy5 NPs were visualized as the largest intracellular clusters. 

**Figure 2:**
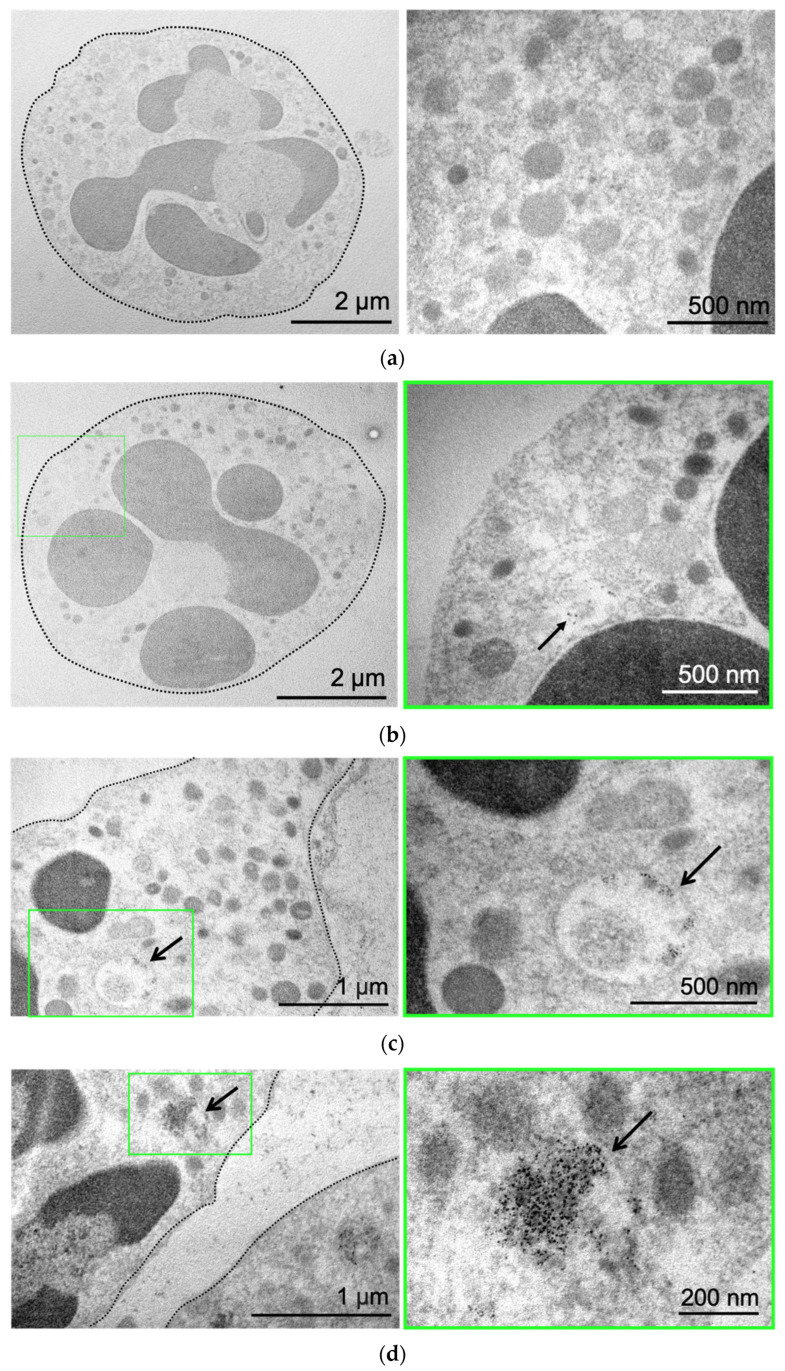
MNP-Cy5 localization in neutrophil blood isolated from the 4T1 tumor-bearing mice: (**a**) microphotographs of control neutrophils cultivated in growth medium; (**b**–**d**) microphotographs of neutrophils incubated with MNPs-Cy5 for 1 h. TEM, green frames indicate areas of cells demonstrated at higher magnification, and arrows point to individual NPs (**b**) or NP conglomerates (**c**) inside vesicles or cytoplasm (**d**).

Next, we used the benefit of MNP electron density to directly visualize them by transmission electron microscopy (TEM). Control neutrophils cultivated in growth medium without NPs are presented in Figure 2a. The cell has a round shape, and the nucleus and mitochondria are clearly visible. Incubation of neutrophils with MNPs-Cy5 did not cause morphological changes in cells (Figure 2b). MNPs were located separately (Figure 2b) or in the form of small conglomerates (Figure 2c), both inside vesicles of various sizes and directly in neutrophils’ cytoplasm (Figure 2d).

Thus, it was found that after 1 h of NP incubation with isolated neutrophils in vitro, all three NP types accumulated inside cells, wherein NPs could be located both in the cytoplasm of neutrophils and inside vesicles that were neither early nor late endosomes, presumably recycling endosomes [38].

**Figure 3:**
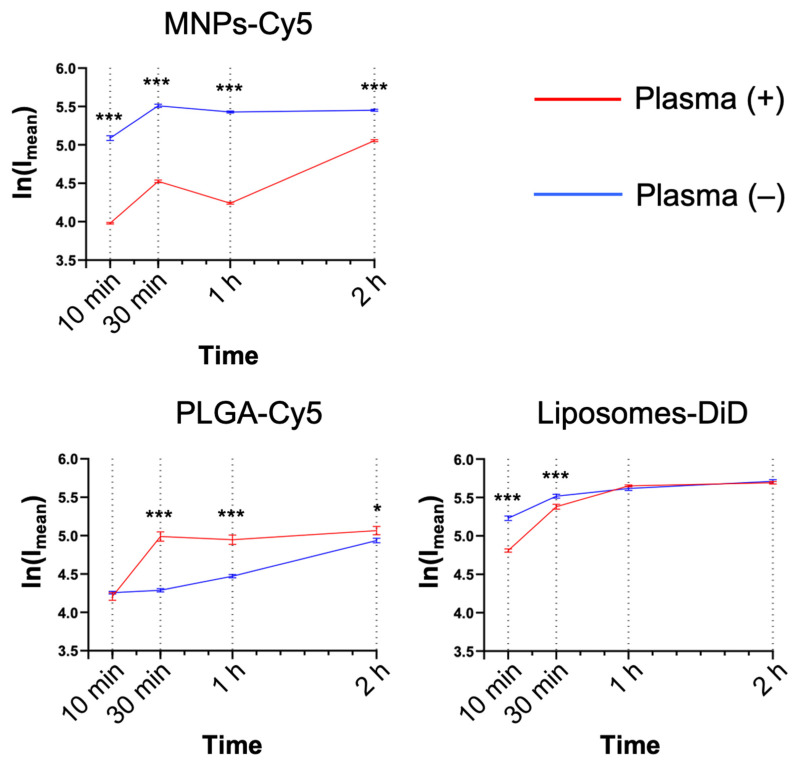
Dynamics of nanoparticle accumulation in neutrophil blood isolated from the 4T1 tumor-bearing mice in the presence and absence of plasma in the culture medium: confocal microscopy results are presented as mean ± SEM, *** *p* < 0.001, * *p* < 0.05 (*t*-test).

Serum factors can significantly affect NP interactions with the cells, both enhancing or preventing NP uptake. For this reason, the NP interaction with neutrophils was studied in the presence or absence of murine plasma (medium:blood plasma = 1:1, *v*:*v*). MNPs-Cy5 interacted with neutrophils in the absence of plasma more efficiently, while PLGA-Cy5 NPs accumulated faster in the presence of plasma (Figure 3), and liposome-DiD interaction with the cells was not affected by the cultivation conditions. The accumulation peak of all NP types in cells was observed after 1 h of co-incubation in most cases, subsequently reaching a plateau.

### 3.2. Nanoparticle Internalization Mechanisms in Neutrophils In Vitro

To determine the mechanisms underlying NP uptake by neutrophils, the following inhibitors were used: (a) pitstop-2 and chlorpromazine to block clathrin-mediated endocytosis (CME); (b) MβCD and nystatin to block caveolin-mediated endocytosis (CavME); and (c) cytochalasin-D and amiloride to block macropinocytosis (MP). First, the inhibitors’ toxicity was studied. It was shown that chlorpromazine, nystatin, cytochalasin-D, and amiloride do not cause significant death of neutrophils at three studied concentrations (Figure S2). MβCD resulted in the death of about 7% of cells at the maximum tested concentration of 5 mM. Pitstop-2 has the most toxic effect on neutrophils, leading to the death of about 20% of cells at a concentration of 50 µM. In addition to the identified negative effect of pitstop-2 on neutrophils at working concentrations (25–50 μM), according to the literature data, this inhibitor has a nonspecific effect on clathrin-independent endocytosis pathways [39]. Moreover, pitstop-2 can lead to disruption of the nuclear membrane integrity [40]. Due to the described nonspecificity and toxicity for neutrophils, it was decided to abandon the use of pitstop-2 and investigate only chlorpromazine as an inhibitor of clathrin-mediated endocytosis. The study was carried out both in the presence of plasma in the culture medium and in its absence.

**Figure 4:**
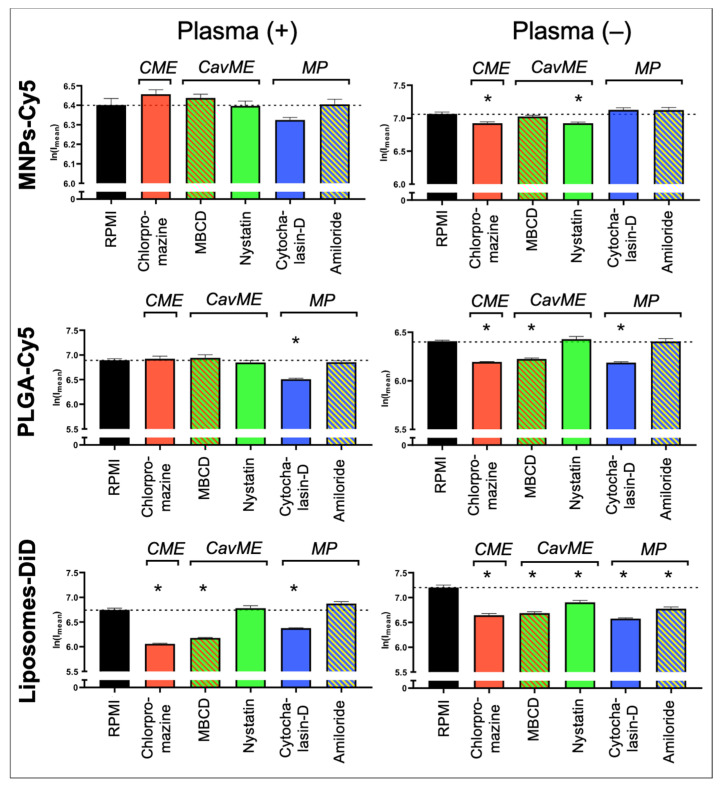
Impact of different endocytosis pathways on nanoparticle uptake by neutrophils: graphs of fluorescence intensity from NPs accumulated in neutrophils (associated with Figures S3–S5). Results are presented as mean ± SEM, * *p* < 0.05 (*t*-test). CME—clathrin-mediated endocytosis; CavME—caveolin-mediated endocytosis; MP—macropinocytosis.

As a result, it was found that none of the inhibitors led to a significant decrease in the MNP-Cy5 uptake by neutrophils when cultivated in the presence of plasma (Figure 4 and Figure S3), wherein, in the absence of plasma, a small but statistically significant (*p* < 0.05, *t*-test) decrease in the MNP-Cy5 internalization was observed when cells were incubated with chlorpromazine or nystatin. Inhibition of PLGA-Cy5 NP endocytosis in the presence of plasma was detected after incubation of neutrophils with cytochalasin-D (Figure 4). It should also be noted that cell cultivation with NPs following cytochalasin-D as well as MβCD and amiloride treatment caused PLGA-Cy5 NP accumulation on the neutrophil membranes without penetration inside the cells (Figure S4). In the absence of plasma, PLGA-Cy5 internalization was inhibited by chlorpromazine, MβCD, and cytochalasin-D. Finally, inhibition of liposome-DiD uptake by neutrophils in the presence of plasma was found after chlorpromazine, MβCD, and cytochalasin-D pre-incubation (Figure 4 and Figure S5). In the absence of plasma, treatment with all investigated inhibitors led to a statistically significant decrease in liposome-DiD engulfment by cells.

The obtained data allow us to conclude that internalization of MNPs-Cy5 by neutrophils can occur due to clathrin- and caveolin-mediated endocytosis. PLGA-Cy5 NPs are characterized by endocytosis dependent on clathrin- and caveolin/raft-mediated mechanisms as well as phagocytosis that indicated a decrease in NP uptake following cytochalasin-D, but not amiloride treatment [41], when incubated without plasma. In the presence of plasma, PLGA-Cy5 NPs are predominantly captured by phagocytosis. Liposomes-DiD, both with or without adding plasma, are taken up by neutrophils due to clathrin- and caveolin/raft-mediated mechanisms of endocytosis, as well as macropinocytosis.

Thus, it was shown that the processes of NP interaction with and uptake by neutrophils are determined not only by the type of NPs but also by plasma components that can modulate endocytosis pathways.

### 3.3. The Role of Plasma Factors and Neutrophil Receptors in Nanoparticle Uptake In Vitro

As PLGA-Cy5 NP accumulation in neutrophils demonstrated the most pronounced dependence on the presence of plasma (Figure 3), we next aimed to determine the impact of plasma factors and surface receptors on NP–cell interactions. For this, we used serum from severe combined immunodeficient (SCID) mice (characterized by low Ig levels [42]), polyinosinic acid (Poly(I)) (class A scavenger receptor-blocking agent [43]), and antibodies to CD16/32 (for blocking Fc receptors [44]). The percentage of cells that captured NPs was analyzed by flow cytometry, and fluorescence intensity from NPs in cells was analyzed by confocal microscopy. 

**Figure 5:**
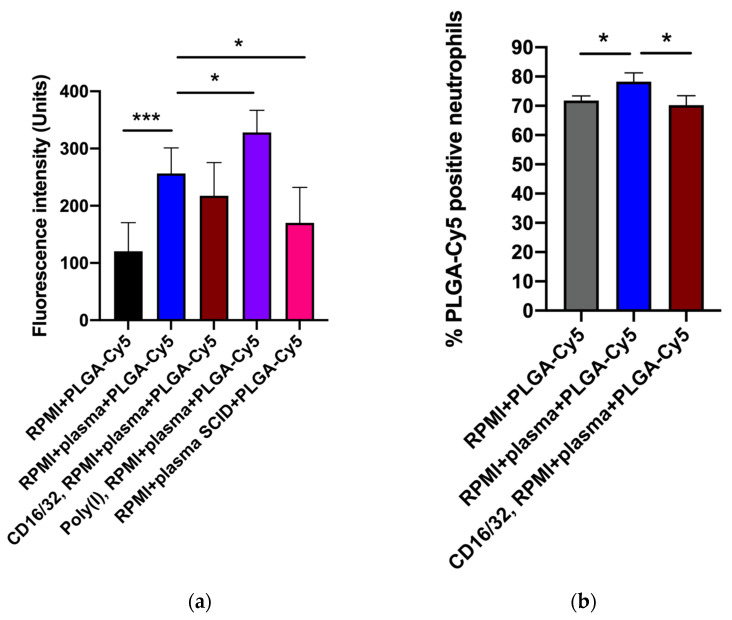
Effectiveness of PLGA-Cy5 NP interaction with isolated neutrophils after 1 h of co-incubation under various conditions: (**a**) fluorescence intensity from Cy5 dye, indicating the efficiency of NP binding by cells, confocal microscopy; (**b**) percentage of neutrophils associated with PLGA-Cy5 NPs relative to all cells of the subpopulation, flow cytometry. Results are presented as mean ± SD, * *p* < 0.05, *** *p* < 0.001 (*t*-test).

The efficiency of PLGA-Cy5 NP interaction with neutrophils in the medium containing plasma was higher than without it (Figure 5 and Figure S6). Figure S6 clearly shows that most of the cells in a subpopulation accumulated NPs in the cytoplasm after an hour of co-incubation in the medium with plasma, whereas without plasma, there were significantly fewer such cells. Moreover, in the latter case, the accumulation of NPs in neutrophils was less pronounced.

Pre-incubation of neutrophils with antibodies to CD16/32 resulted in a small but statistically significant decrease in the efficiency of PLGA-Cy5 NP interactions with cells (Figure 5). Scavenger receptor-blocking with Poly(I) did not inhibit NPs binding by neutrophils (Figure 5a). Finally, cultivation of cells in a medium supplemented with plasma from SCID mice led to a decrease in the Cy5 signal intensity in neutrophils.

Thus, the data obtained clearly demonstrated that plasma presence in the culture medium had a significant effect on PLGA-Cy5 NP binding by neutrophils, increasing it. The absence of IgG/IgM as well as Fc receptor-blocking neutralized this effect, reducing the efficiency of NP interaction with cells. It can be concluded that immunoglobulins lead to the formation of NP agglomerates, which are subsequently captured by neutrophils through the Fc receptors.

## 4. Discussion

In the current study, the investigation of three NP types’ (liposomes, PLGA, and magnetic NPs) interaction with murine neutrophils was continued [34]. 

All NPs were shown to penetrate neutrophils isolated from the tumor-bearing mice and localize directly in cell cytoplasm or in vesicles of different sizes. However, after an hour of co-incubation, NPs were no longer inside early or late endosomes, presumably in endolysosomes or phagosomes. The pathways of NP endocytosis by neutrophils in vitro were studied using an inhibitor assay [16,45,46]. Chlorpromazine, which disrupts the formation of clathrin coating around developing endosomes, was used to investigate clathrin-dependent endocytosis [16]. MβCD and nystatin, depleting cell membrane cholesterol, lead to inhibition of caveolin/raft-dependent types of endocytosis. Finally, cytochalasin-D, which disrupts actin polymerization, as well as amiloride, which blocks the sodium/proton pump causing the acidification of the cytoplasm near the plasma membrane and disruption of actin polymerization, inhibit the processes of phago- and macropinocytosis. Thus, for small negatively charged MNPs-Cy5, all types of endocytosis were found to participate in NP uptake. The absence of a significant decrease in MNPs-Cy5 engulfment after incubation with inhibitors may be due to the fact that non-blocked pathways compensated for the lack of endocytosis through an inhibited mechanism. Interestingly, Portilla et al. also demonstrated that positively charged 3-aminopropyl-trietoxysilane (APS)-coated MNPs with hydrodynamic sizes of 122 (±1.3) nm in distilled water and 1293 nm in culture medium containing 10% FBS were internalized through all endocytic pathways by RAW264.7 cells. On the contrary, the uptake of negatively charged dimercaptosuccinic acid (DMSA)-coated MNPs with hydrodynamic sizes of ~83 (±1.0) nm in water and 117 nm in growth medium was limited to clathrin- and caveolin-dependent mechanisms [47]. However, in Pan02 cell culture, the APS-MNPs were engulfed through macropinocytosis, while the DMSA-MNPs penetrated via all endocytic pathways. Svitkova et al. showed that negatively charged PEG-MNPs with a hydrodynamic size of (76.0 ± 2.52) nm were taken up via caveolin-mediated endocytosis or lipid rafts by A549 cells, but bovine albumin-coated MNPs with similar physicochemical parameters were penetrated by clathrin-mediated endocytosis [48]. We also revealed that liposomes-DiD were engulfed by neutrophils due to clathrin- and caveolin/raft-mediated mechanisms of endocytosis, as well as macropinocytosis. But unlike MNPs-Cy5, inhibition of one of these mechanisms led to a pronounced decrease in the NP accumulation efficiency, which was not compensated by the work of other pathways. These three types of endocytosis for liposomes were described in different studies [49]. The pathway of NP internalization could vary and even change depending on cultivation conditions and cells. For example, preincubation of DOTAP:DOPC:DOPE:DC-cholesterol liposomes with human plasma resulted in the protein corona formation and transition of the macropinocytotic uptake into clathrin-mediated endocytosis. Since the culture conditions, cell type, shape, and coating of NPs were the same in our study, the difference in the contribution of endocytosis pathways in MNP-Cy5 and liposome-DiD uptake by neutrophils may be related to the particle size. PLGA-Cy5 NP endocytosis pathways differed depending on the cultivation conditions. Incubation without plasma resulted in clathrin- and caveolin/raft-mediated mechanisms as well as phagocytosis, and addition of plasma increased the role of phagocytosis. We assume that this is due to the formation of fairly large conglomerates of NPs in a medium containing plasma (Figure S1). Since this type of NP is not coated with PEG, the presence of plasma leads to the formation of a protein corona and, as a consequence, such conglomerates [50]. As noted above, these conglomerates are precisely accessible for phagocytosis [14]. Thus, all endocytic pathways, such as clathrin- and caveolin/raft-mediated, macropinocytosis, and phagocytosis, can be involved in the process of three investigated NP uptake, but their contribution varies for each type of NP, which differ in size. Moreover, the presence or absence of plasma in culture medium is also an important factor for the determination of the way by which NPs enter the cells. This is especially true for NPs that tend to form conglomerates upon contact with plasma. Therefore, the choice of the right surface modification can prevent the formation of large NP conglomerates. For example, coating with BSA or autologous serum proteins can help to maintain colloid stability in the presence of serum and, as a result, decrease the phagocytosis of NP conglomerates, leading to prolonged blood circulation [51,52,53].

In order to identify which aspects associated with the presence of plasma in a medium may be involved in NP endocytosis, we investigated the role of IgG/IgM. For this purpose, neutrophils were cultured in a medium supplemented with serum from SCID mice with very low Ig levels [42]. The decreased level of IgG/IgM resulted in a reduced number of cells interacting with PLGA-Cy5 NPs. IgG receptor-blocking demonstrated the same effect. We assume that PLGA-Cy5 NPs can be captured by neutrophils through the Fc receptors in the presence of immunoglobulins. For a more detailed analysis of the plasma protein role in NP uptake by neutrophils, other components, such as the complement, should be studied further.

Other receptors on the surface of neutrophils that can take part in the process of NP uptake are scavenger receptors. These are membrane-associated pattern recognition receptors that act as phagocytic receptors, mediating direct uptake of pathogenic microbes and/or their products [54]. Yang et al. demonstrated that modification of gold NPs with bacterial lipopolysaccharide increased their internalization by neutrophils via class A scavenger receptors [55]. Wang et al. showed that a class A1 scavenger receptor is a receptor for carboxylated multi-walled carbon nanotubes (cMWNTs) coated with Pluronic^®^ F-108 [56]. Huynh et al. continued this investigation and found that BSA-coated cMWNTs accumulated in RAW 264.7 macrophages due to a class A1 scavenger receptor two-times more effective than the same NPs without corresponding coating [57]. However, our data demonstrated that the binding of PLGA-Cy5 NPs by neutrophils is not dependent on scavenger receptors. This may be due to the fact that, unlike macrophages, neutrophils contain fewer scavenger receptors [58]. Moreover, for neutrophils, it was shown that their internalization of polymersomes depends on scavenger receptors class B [59], while Poly(I) blocks class A scavenger receptors [43]. Thus, to obtain a more complete view on the neutrophil interaction with different types of NPs, it is necessary to continue the study using other research approaches. Understanding the mechanisms of NP binding by neutrophils can increase the efficiency of ex vivo cell loading with NPs. This, in turn, is important for further enhancement of neutrophil-mediated nanodrug delivery to the tumor. The effectiveness of this strategy has already been demonstrated by two groups of researchers in mouse, rat, and human glioma models [60,61]. The obtained results can help to increase the effectiveness of neutrophil-based nanodrug delivery to neoplasms due to the choice of a specific type of nanocarrier for antitumor drug loading.

## 5. Conclusions

In the present study, the pathways of liposome, PLGA, and magnetite NP internalization by neutrophils isolated from the blood of 4T1 tumor-bearing mice were investigated. Since blood plasma factors significantly affect NP interactions with the cells, different cultivation conditions were examined. It was revealed that small 35 nm MNPs-Cy5 can penetrate neutrophils by caveolin-/clathrin-mediated endocytosis as well as micropinocytosis, both in the presence or absence of plasma. In the meantime, plasma factors affected PLGA-Cy5 NP internalization pathways. Incubation without plasma resulted in a clathrin- and caveolin/raft-mediated uptake as well as phagocytosis, while in plasma-containing media, PLGA-Cy5 NP internalization was limited to phagocytosis only. Finally, liposomes-DiD were engulfed via clathrin-, caveolin/raft-mediated mechanisms, and macropinocytosis in both cultivating conditions. For PLGA-Cy5 NPs, it was also found that the internalization depends on IgG/IgM presence in the medium as well as Fc, but not scavenger, receptors on the cell surface. These findings can help increase the efficiency of ex vivo cell loading with NPs and, as a result, enhance the neutrophil-mediated nanodrug delivery to the tumor.

## Data Availability

The data presented in this study are available on request from the corresponding author. The data are not publicly available due to privacy.

## Supplementary Materials

The following supporting information can be downloaded at https://www.mdpi.com/article/10.3390/biomedicines12102180/s1, Figure S1: Dynamics of NP hydrodynamic (HD) size during 2-day incubation in 10 mM PBS (pH 7.4) at 4 °C or growth medium containing 10% FBS at 37 °C. Results are presented as mean ± SD. The stability of liposomes-DiD is presented in [37]; Figure S2: Cytotoxicity of endocytosis inhibitors for neutrophil blood isolated from the 4T1 tumor-bearing mice. LDH test. Results are presented as mean ± SD; Figure S3: Accumulation of MNPs-Cy5 in neutrophil blood isolated from the 4T1 tumor-bearing mice, incubated with different endocytosis inhibitors and then with NPs in the presence or absence of plasma in a culture medium; microphotographs of cells stained with antibodies to Ly6G (blue), confocal microscopy. MNPs-Cy5 are red; Figure S4: Accumulation of PLGA-Cy5 NPs in neutrophil blood isolated from the 4T1 tumor-bearing mice, incubated with different endocytosis inhibitors and then with NPs in the presence or absence of plasma in a culture medium; microphotographs of cells stained with antibodies to Ly6G (blue), confocal microscopy. PLGA-Cy5 NPs are red; Figure S5: Accumulation of liposomes-DiD in neutrophil blood isolated from the 4T1 tumor-bearing mice, incubated with different endocytosis inhibitors and then with NPs in the presence or absence of plasma in a culture medium; microphotographs of cells stained with antibodies to Ly6G (blue), confocal microscopy. Liposomes-DiD are red; Figure S6: Interaction of PLGA-Cy5 NPs with neutrophil blood isolated from the 4T1 tumor-bearing mice after 1 h of co-incubation under various conditions; microphotographs of cells stained with antibodies to Ly6G (blue), CD45 (green), and CD11b (white), confocal microscopy. PLGA-Cy5 NPs are red.
