# Supplementary material for: The Internalization Pathways of Liposomes, PLGA, and Magnetic Nanoparticles in Neutrophils
Source: Biomedicines. 2024 Sep 25;12(10):2180. doi: 10.3390/biomedicines12102180 (PMC11505478; doi:10.3390/biomedicines12102180)
Supplement: Supplementary file 1 [file biomedicines-12-02180-s001.zip › biomedicines-3194135-supplementary.pdf]

Supplementary Information

Results

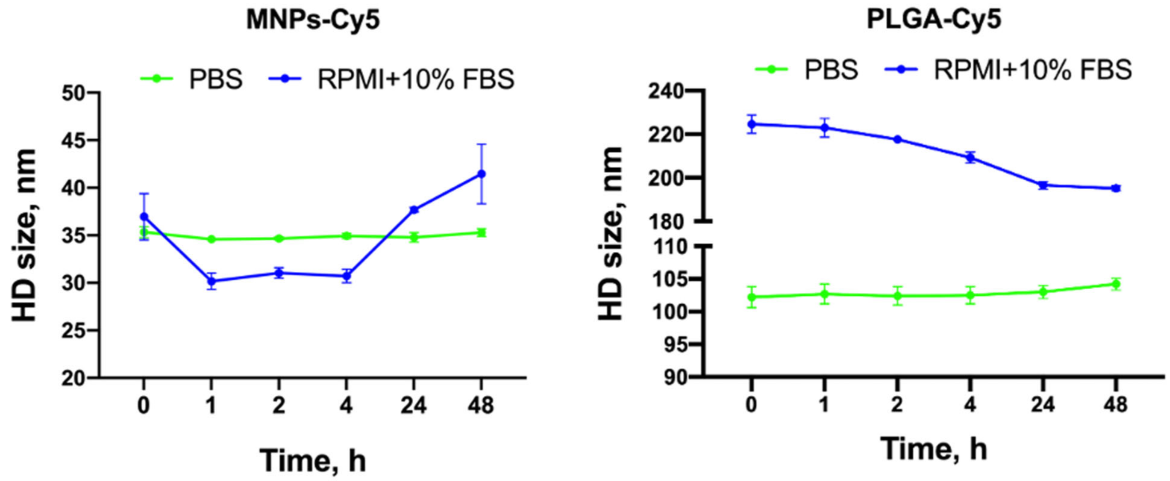

Figure S1. Dynamics of NP hydrodynamic (HD) size during 2 days incubation in 10 mM PBS (pH 7.4) at 4˚C or growth medium containing 10% FBS at 37˚C. Results are presented as mean ± SD. The stability of liposomes-DiD was presented in [37].

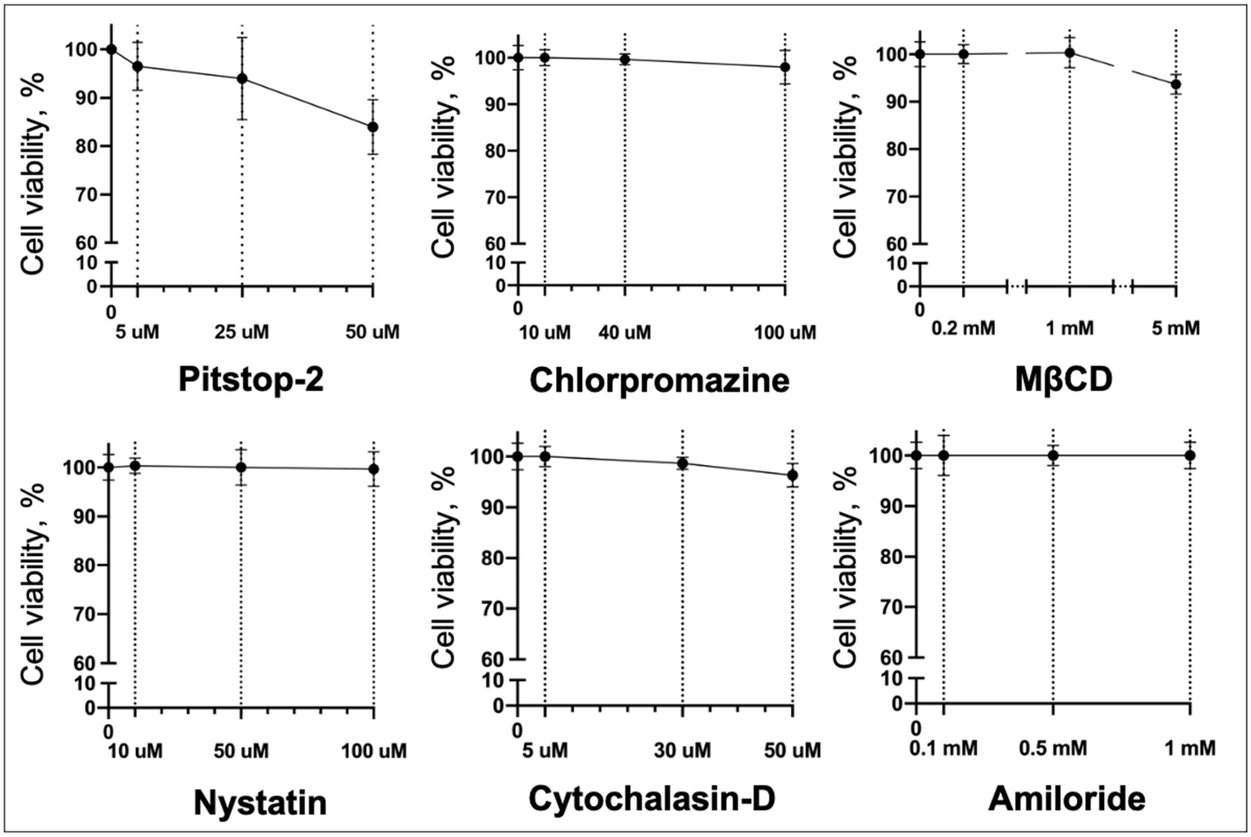

Figure S2. Cytotoxicity of endocytosis inhibitors for neutrophils blood isolated from the 4T1 tumor-bearing mice. LDH test. Results are presented as mean ± SD.

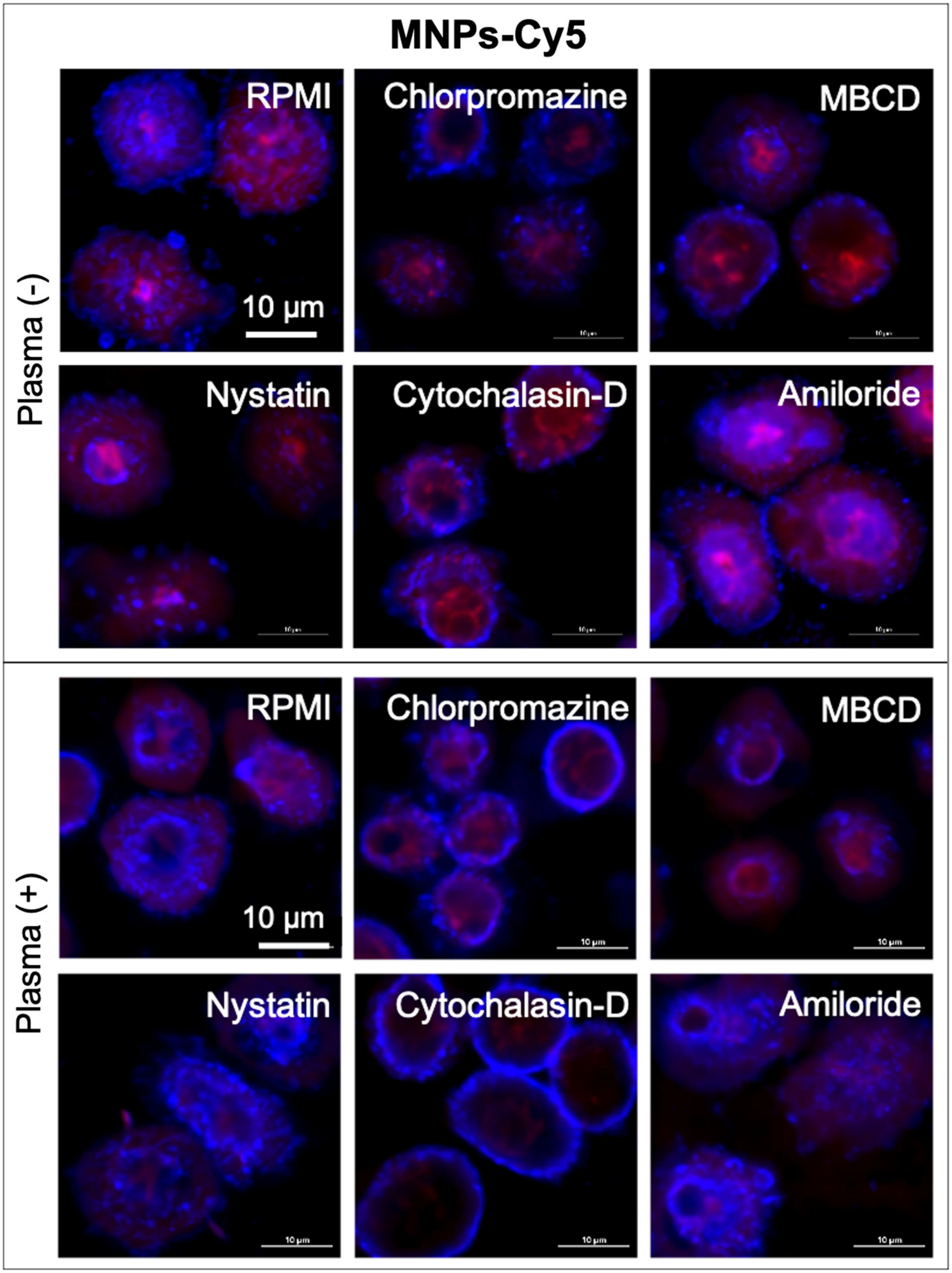

Figure S3. Accumulation of MNPs-Cy5 in neutrophils blood isolated from the 4T1 tumor-bearing mice, incubated with different endocytosis inhibitors, and then with NPs in the presence or absence of plasma

in culture medium: microphotographs of cells stained with antibodies to Ly6G (blue), confocal microscopy. MNPs-Cy5 are red.

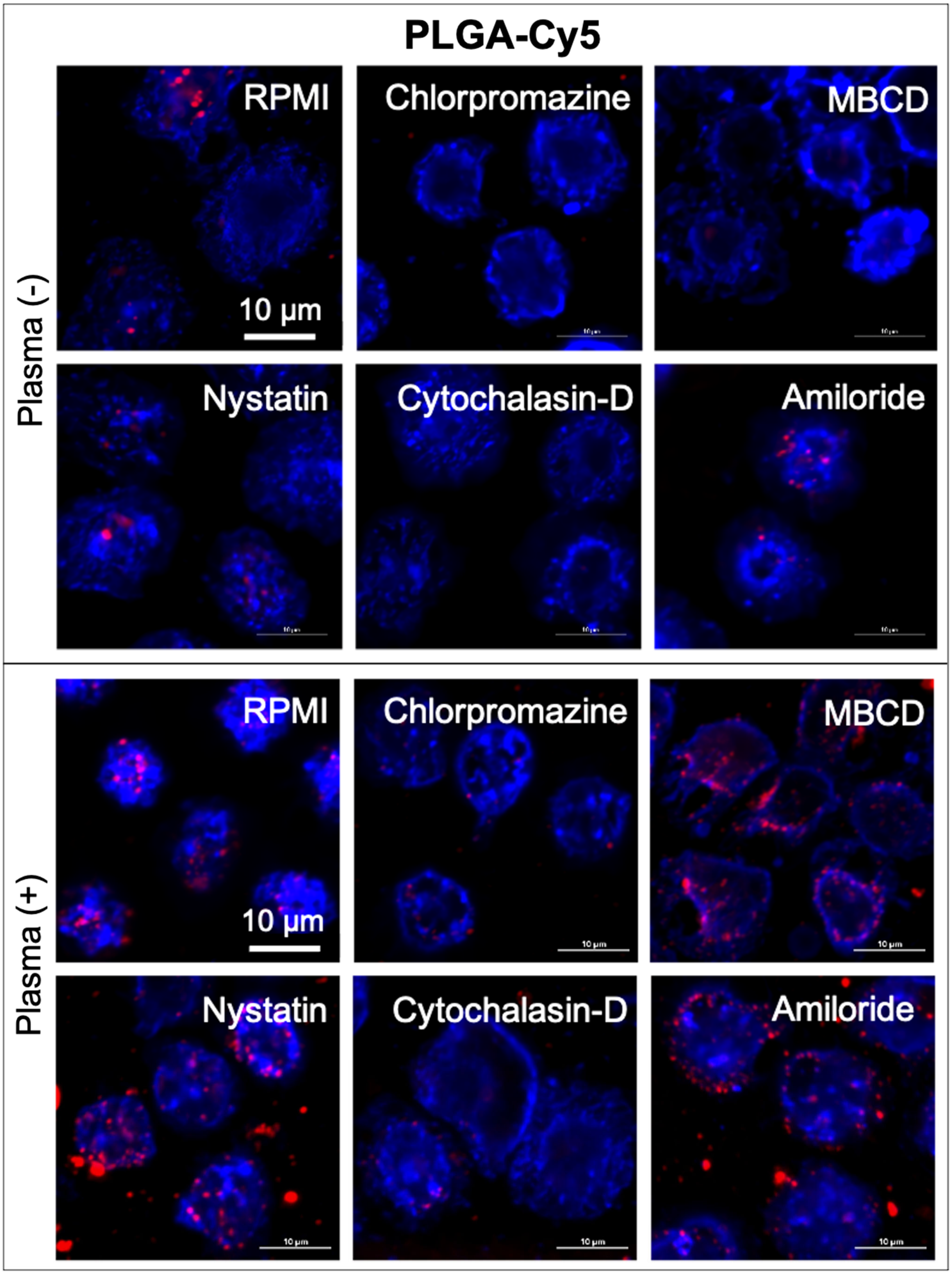

Figure S4. Accumulation of PLGA-Cy5 NPs in neutrophils blood isolated from the 4T1 tumor-bearing mice, incubated with different endocytosis inhibitors, and then with NPs in the presence or absence of

plasma in culture medium: microphotographs of cells stained with antibodies to Ly6G (blue), confocal microscopy. PLGA-Cy5 NPs are red.

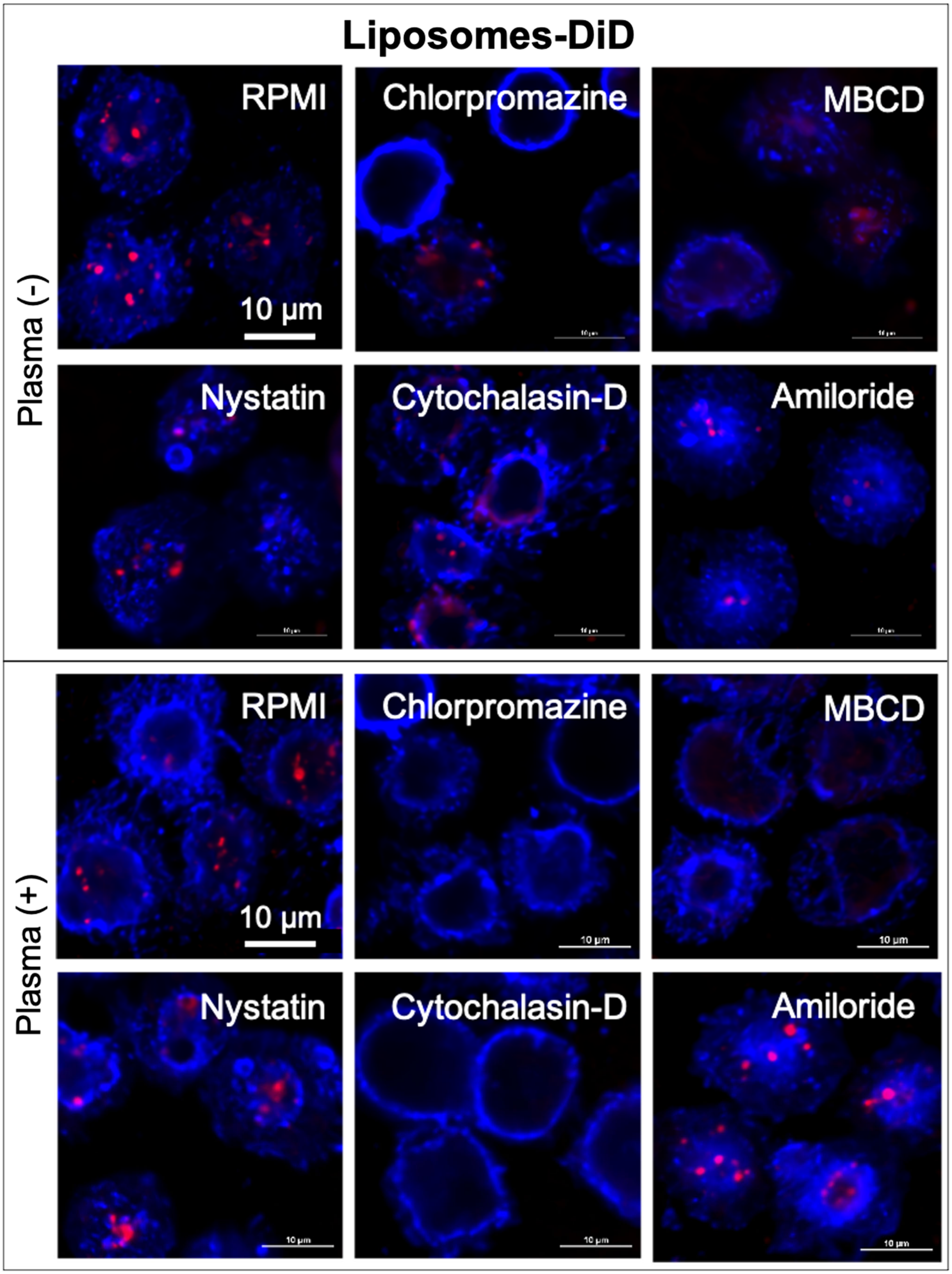

Figure S5. Accumulation of liposomes-DiD in neutrophils blood isolated from the 4T1 tumor-bearing mice, incubated with different endocytosis inhibitors, and then with NPs in the presence or absence of

plasma in culture medium: microphotographs of cells stained with antibodies to Ly6G (blue), confocal microscopy. Liposomes-DiD are red.

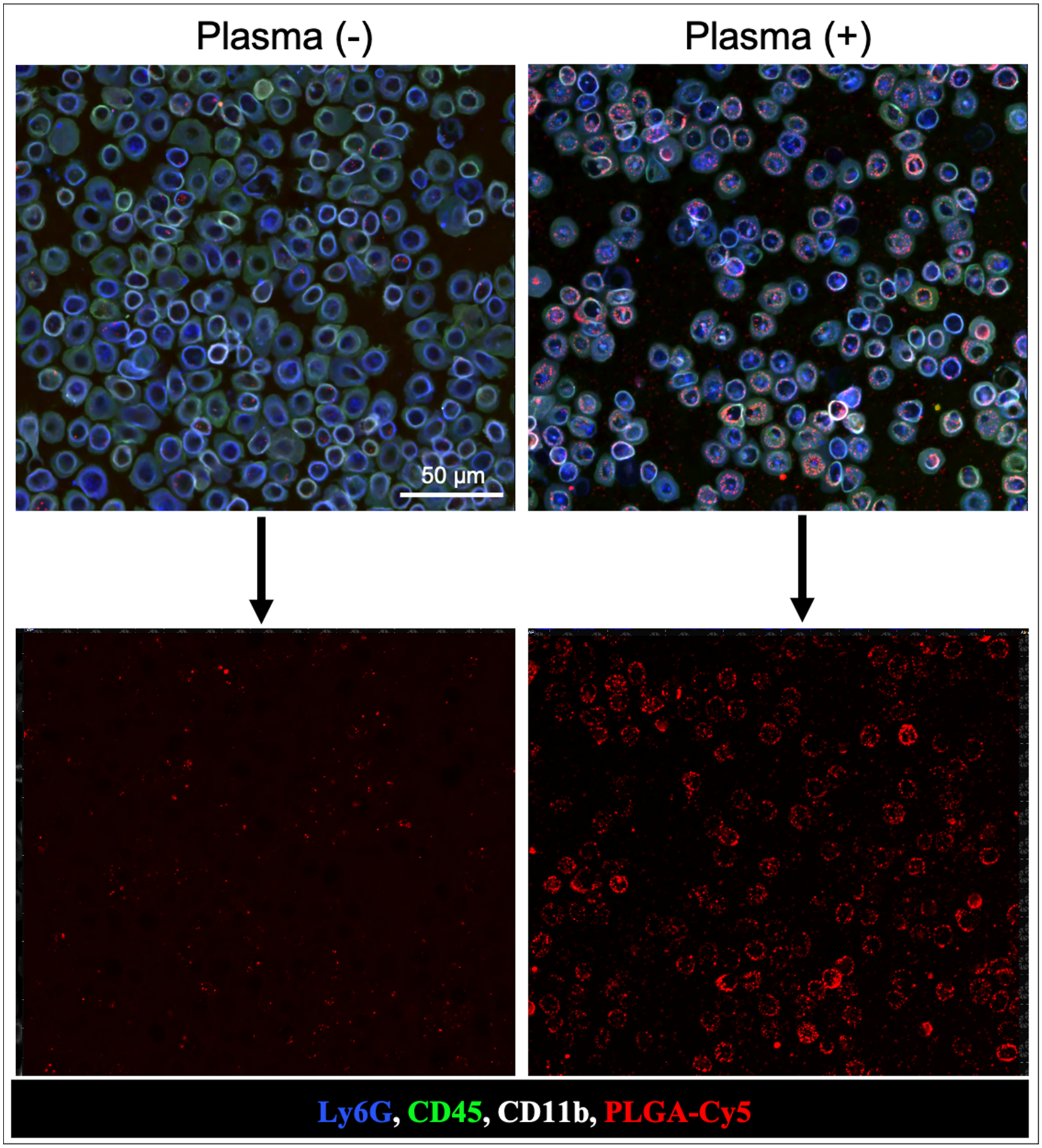

Figure S6. Interaction of PLGA-Cy5 NPs with neutrophils, blood isolated from the 4T1 tumor-bearing mice, after 1 hour of co-incubation under various conditions: microphotographs of cells stained with antibodies to Ly6G (blue), CD45 (green), and CD11b (white), confocal microscopy. PLGA-Cy5 NPs are red.

Materials and methods

1. Nanoparticles' synthesis

1.1. MNPs-Cy5

Magnetic nanoparticles were obtained via the thermal decomposition of iron (III) acetylacetonate (10.48 g) in benzyl alcohol (220 mL). For the MNPs' (suspension of 80 mg in a 30 mM aqueous NaOH solution) coating with protein, an aqueous solution of human serum albumin (HSA) (8 mg/mL, 20 mL) was used. For coating the obtained NPs with polyethylene glycol (PEG), 5 mg of MNPs-HSA was dissolved in PBS buffer (pH 7.4) (to an Fe^{3+} concentration of 0.95 mg/mL). Then, 266 µL of N-hydroxysuccinimide (NHS) solution in PBS (10 mg/mL) and 457 µL of 1-ethyl-3-(3-dimethylaminopropyl)carbodiimide (EDC) solution in PBS (10 mg/mL) were added. The reaction mixture was incubated with stirring for 10 min, after which, 470 µL of polyethylene glycol hydrochloride salt NH_2-PEG-OH solution in dH_2O (10 mg/mL) was added. The incubation time was 1 hour. The resulting MNPs-HSA-PEG were separated from the PEG excess via gel filtration using a NAP-10 column (Sephadex G25, eluent PBS, GE Healthcare Bio-Sciences, Chicago, IL, USA). For the MNPs-HSA conjugation with the fluorescent dye Cyanine 5 amine (Cyanine5, an amine derivative, Lumiprobe, Hanover, Germany), 126 µL of EDC solution in PBS (10 mg/mL) and 189 µL of NHS solution in PBS (10 mg/mL) were added to 9 mL of the MNPs (3.5 mg Fe^{3+}/mL). The reaction mixture was incubated for 15 min at room temperature. After that, 315 µL of Cy5 solution (1 mg/mL in DMSO) was added. Incubation with a fluorescent label was carried out for 15 h at room temperature. The excess label was removed using a mini-column PD-10 (Sephadex G25, eluent-PBS).

1.2. PLGA-Cy5 NPs

For the PLGA-Cy5 NPs' synthesis, a polymer preliminarily modified with a Cy5 fluorescent label was used. The fluorescent dye Cyanine 5 amine was covalently linked to the carboxyl end group of a lactic-glycolic acid copolymer (PLGA, Resomer® 502H, Mw 7–17 kDa, $\eta = 0.21$; Evonik Röhm GmbH, Weiterstadt, Germany) by the means of the carbodiimide method in 2 steps. Weighed polymer (1.939 g), EDC (6.6 mg [42.8 µmol]), NHS (2.5 mg), and diisopropylamine (DIEA) (22 mL) were dissolved in methylene chloride. The reaction mixture was stirred overnight at room temperature in the dark. Then, a dye solution (Cy5 2.8 mg (4.28 µmol)) in methylene chloride and, additionally, EDC (1.7 mg (8.5 µmol)) were added. The reaction was carried out for 48 h at room temperature in the dark. The reaction mixture was washed three times with a mixture of equal volumes of water and methanol to remove water-soluble by-products and unreacted starting reagents. The organic phase was separated in a separating funnel, dried over anhydrous sodium sulfate, and evaporated on a rotary evaporator. The

resulting precipitate was dissolved in ethyl acetate (approximately 10 mL of ethyl acetate per 1 g of polymer) and added to a tenfold volume of hexane to precipitate the polymer, filtered, and dried in a desiccator. The formation of the conjugate and the absence of impurities were determined using thin layer chromatography (TLC) on plates (eluent methylene chloride:methanol:water 6.5:2.5:0.4, v/v). The content of Cy5 in the conjugate was measured spectrophotometrically (λ_{abs} = 630 nm) Shimadzu UV-1800, and fluorescence spectra were obtained using a Shimadzu RF-6000 spectrofluorimeter. Fluorescently labeled NPs were obtained through the method of simple emulsions (oil/water). Weighed portions of PLGA polymers (300 mg of the original and 300 mg of the fluorescently modified one) were dissolved in 12 mL of methylene chloride. The resulting solution was added to 60 mL of a 1% solution of polyvinyl alcohol (9-10 kDa) in distilled water and homogenized on an UltraTurrax T18 mechanical disperser with a G10 nozzle for 2 min at 23,600 RMP with cooling; then, on a high-pressure homogenizer Microfluidizer® M-110P (1.5 min at 15,000 psi), residual organic solvent was removed under vacuum. Later, the nanosuspension was filtered, a cryoprotectant (2.5% D-mannitol) was added, poured into vials (1.25 mL each), and lyophilized (Alpha 2-4 LSCplus, Martin Christ GmbH, Osterode am Harz, Germany). Freeze-dried samples were stored at 4°C.

1.3. Liposomes-DiD

The liposomes were obtained via thin lipid film hydration. Egg lecithin (10 mg) (PanReac AppliChem, Darmstadt, Germany), DSPE-PEG2000 (3.4 mg) (Avanti Polar Lipids, Alabaster, AL, USA), cholesterol (3.4 mg) (Avanti Polar Lipids, USA), and DiD (1,1′-dioctadecyl-3,3,3′,3′-tetramethylindodicarbocyanine, 4-chlorobenzenesulfonate salt) fluorescent dye (15 µg) (Invitrogen, Waltham, MA, USA) were dissolved in 1 mL of chloroform at the bottom of a round bottom flask. To form a thin lipid film, chloroform was removed on a rotary evaporator (45°C, 120 rpm, 200 Pa). The formed lipid film was hydrated at room temperature by adding 1 mL of 10 mM sodium phosphate buffer (PBS, pH 7.4). The lipids were dispersed by stirring for 15 min and then by sonication in an ultrasonic bath for 1 min (22 kHz). To obtain a homogeneous dispersion of liposomes, the solution was sequentially extruded through carbon membranes with pore sizes of 0.4, 0.2, and 0.1 µm, respectively. For the experimental work *in vitro* and *in vivo*, fluorescent liposomes were prepared with a final lipid concentration of 16.8 mg/mL in 10 mM PBS. The concentration of DiD in the composition of the prepared liposomes solution was determined using spectrophotometry at an absorption wavelength (λ_{abs}) equal to 644 nm (εDiD = 244,000 L/cm/mol).

2. Nanoparticles' stability assay

The stability of MNPs-Cy5 and PLGA-Cy5 was examined by incubating the NPs in 10 mM PBS (pH 7.4) at 4˚C and in RPMI-1640 containing 10% FBS (gibco) at 37˚C during 2 days. At time points

(0, 1, 2, 4 hours and 1, 2 days) aliquots were analyzed by dynamic light scattering (DLS) using a Malvern Zetasizer Nano ZS (Malvern Instruments, MA, USA) to determine HD size and polydispersity index (PDI).

3. LDH assay

Isolated neutrophils were seeded in a 96-well plate (Corning, Somerville, MA, USA) at a concentration of $1{,}5 \times 10^5$ cells per well in triplicate for each endocytosis inhibitor and concentration. The cells were left for 1 hour at 37°C. Then, pitstop-2 (Abcam, USA), chlorpromazine (aminazine, Valenta, Russia), MβCD (Sigma, New York, NY, USA), nystatin (Sigma), cytochalasin-D (Sigma), or amiloride (Sigma) were added to the medium, and the cells were incubated for 1 hour under the same conditions. Neutrophils cultured in the growth medium (Spontaneous LDH activity) and in the presence of a lysis buffer (Maximum LDH activity) included in the commercial LDH Cytotoxicity Assay Kit (ThermoFisher Scientific) were used as controls. The assay for assessing the release of LDH was carried out in accordance with the manufacturer's instructions. After the incubation time, 50 μL of the medium from the cells was transferred into the wells of a new 96-well plate. In total, 50 μL of the reaction mixture from the commercial kit was added to the same wells, pipetted, and left for 30 min at room temperature in the darkness. Finally, 50 μL of stop solution was added and the absorbance of the obtained samples was analyzed on a plate analyzer (EnSpire 2300 Multilabel Reader, PerkinElmer, Waltham, MA, USA) at 490 and 680 nm. The percentage of inhibitor cytotoxicity was calculated using the following formula:

$$\%\ Cytotoxicity = \frac{100 * (\text{«}Compound\ treated\ LDH\ activity\text{»} - \text{«}Spontaneous\ LDH\ activity\text{»})}{(\text{«}Maximum\ LDH\ activity\text{»} - \text{«}Spontaneous\ LDH\ activity\text{»})}$$

Survival was recalculated as (100% - % cytotoxicity).
